# Tissue-specific control of latent CMV reactivation by regulatory T cells

**DOI:** 10.1371/journal.ppat.1006507

**Published:** 2017-08-10

**Authors:** Maha Almanan, Jana Raynor, Allyson Sholl, Mei Wang, Claire Chougnet, Rhonda D. Cardin, David A. Hildeman

**Affiliations:** 1 Department of Pediatrics, University of Cincinnati College of Medicine, Division of Immunobiology, Children’s Hospital Medical Center, Cincinnati, OH, United States of America; 2 Department of Pediatrics, University of Cincinnati College of Medicine, Division of Infectious Diseases, Children’s Hospital Medical Center, Cincinnati, OH, United States of America; 3 Department of Pathobiological Sciences, School of Veterinary Medicine, Louisiana State University, Baton Rouge, LA, United States of America; University of Wisconsin-Madison, UNITED STATES

## Abstract

Cytomegalovirus (CMV) causes a persistent, lifelong infection. CMV persists in a latent state and undergoes intermittent subclinical viral reactivation that is quelled by ongoing T cell responses. While T cells are critical to maintain control of infection, the immunological factors that promote CMV persistence remain unclear. Here, we investigated the role of regulatory T cells (Treg) in a mouse model of latent CMV infection using Foxp3-diphtheria toxin receptor (Foxp3-DTR) mice. Eight months after infection, MCMV had established latency in the spleen, salivary gland, lung, and pancreas, which was accompanied by an increased frequency of Treg. Administration of diphtheria toxin (DT) after establishment of latency efficiently depleted Treg and drove a significant increase in the numbers of functional MCMV-specific CD4+ and CD8+ T cells. Strikingly, Treg depletion decreased the number of animals with reactivatable latent MCMV in the spleen. Unexpectedly, in the same animals, ablation of Treg drove a significant increase in viral reactivation in the salivary gland that was accompanied with augmented local IL-10 production by Foxp3-CD4+T cells. Further, neutralization of IL-10 after Treg depletion significantly decreased viral load in the salivary gland. Combined, these data show that Treg have divergent control of MCMV infection depending upon the tissue. In the spleen, Treg antagonize CD8+ effector function and promote viral persistence while in the salivary gland Treg prevent IL-10 production and limit viral reactivation and replication. These data provide new insights into the organ-specific roles of Treg in controlling the reactivation of latent MCMV infection.

## Author summary

Cytomegalovirus (CMV) infection in both mice and humans is normally initially contained by a vigorous adaptive immune response that drives the virus into latency in multiple tissues. However, the immunologic mechanisms that control latency are not well understood. In this report, we have examined the role of regulatory T cells (Treg) in a mouse model of CMV infection. Interestingly, depletion of regulatory T cells had profound consequences on MCMV latent infection, depending upon the tissue. In the spleen, Treg depletion enhanced CD8+ T cell responses and reduced reactivatable latent infection from the spleen. In striking contrast, in the salivary gland, Treg depletion enhanced the production of IL-10 from CD4+ T cells as well as viral reactivation. Thus, Treg play divergent and tissue specific roles in controlling MCMV reactivation from latency.

## Introduction

The immune system has evolved multiple innate and adaptive strategies to control pathogens[1]. Likewise, in order to ensure their persistence, pathogens have developed sophisticated and elaborate mechanisms to avoid the host immune system establishing latent infections that are never cleared from the host [2–7]. Human cytomegalovirus (HCMV) and its murine homolog (MCMV) are well-studied examples of pathogens that have developed multiple means to establish latency [8–10]. MCMV is a reasonable model for HCMV as it shares multiple biological characteristics and significant homology to the genome of HCMV[11]. A large number of HCMV and MCMV genes are involved in modulating innate and adaptive host immune responses [12–15]. During primary infection, these viruses vigorously replicate and disseminate by infecting many cell types, including epithelial, endothelial, smooth muscle, and connective tissue cells, as well as specialized parenchymal cells in multiple tissues [16].

Primary CMV infection is well controlled by a robust early NK cell response followed by CD4+ and CD8+ T cell responses that ultimately results in control of virus replication, although the virus is not eliminated and persists for the lifetime of the host [17–20]. Interestingly, prior work shows that the control of lytic virus in different tissues requires distinct immune cell populations [21–25]. In the spleen, epitope-specific CD8+ T cells are sufficient to control acute MCMV infection [26]. Whereas in the salivary gland (SG), CD4+ T cells and, in particular their production of IFN-γ, are crucial for terminating lytic viral replication. Further, IL-10R blockade or the absence of CD4+ cell-derived IL-10 enhanced the accumulation of IFN-γ–producing CD4+ T cells and inhibited MCMV persistence in the SG [21, 22, 25, 27–29]. This role of CD4+ T cells in the SG is critical, as there appears to be a reservoir of MCMV in non-hematopoietic cells within the SG that downregulate MHC class I and become resistant to CD8+ T cell killing [22]. However, recent work showed that CD8+ T cells can play a role in controlling MCMV in the SG after local re-infection [30]. The likely explanation for this differential role of CD8+ T cells during re-infection is because of the higher expression of class I MHC in acutely infected cells in contrast to cells harboring latent virus. Regulatory T cells (Treg) have also been shown to contribute to immune-mediated control of acute MCMV infection. *In vitro*, Treg were shown to suppress the function of MCMV-specific CD8+T cells via secretion of TGF-ß [31]. *In vivo*, during acute MCMV, Treg depletion resulted in enhanced MCMV-specific T cell responses and decreased viral load [32]. Nonetheless, following the termination of lytic infection, the virus persists in a latent state in which viral genomes are present in the absence of replicating virus.

MCMV establishes latency in a number of tissues similar to HCMV, including the spleen, lungs, and bone marrow [33–36]. This latent form of HCMV drives a substantial amount of morbidity when it reactivates in immune suppressed individuals (e.g. aged individuals, persons with HIV infection, transplant patients) [37–39]. During latent infection, HCMV persists in CD34+ hematopoietic stem cells as well as more committed myeloid lineage progenitor cells, monocytes, and macrophages [40–44]. Both myeloid lineage cells [33, 45], as well as non-hematopoietic cells such as endothelial cells in many organs are considered cellular sites of latent infection for MCMV [33, 46, 47].While a fair amount is known regarding immune mechanisms controlling acute MCMV infection, significantly less is known about immune mechanisms contributing to the control of latent MCMV infection. It has been reported that once latency is established, the cooperative function of lymphocytes including NK, CD4+, CD8+ T cells and CMV-specific antibodies prevent the production of lytic virus from latent pools in the spleen and lungs and SG [48]. In most visceral organs, CD8+ T cells play a crucial role in preventing the emergence of lytic viral replication. For example, CD8+ T cells maintain latency by epitope-specific sensing of transcriptional reactivation in the lungs and killing these cells [49]. Similar to their role in controlling acute infection in the SG, CD4+ T cell production of IFN-γ is critical to prevent lytic virus production from latently infected cells [22, 50, 51].

During latency, there is substantial epigenetic suppression of viral immediate-early genes that must be overcome by cellular signals to exit from latency [42, 44, 52]. Two models have been proposed for MCMV reactivation during latency. First, a two-step model proposed by Hummel et al. [53] in which an inflammatory immune response can drive the activation of the major immediate-early (MIE) gene, initiating reactivation from latency. The second step requires an immune-suppressive environment, such as that which occurs during γ-irradiation or immune-deficiency. This immune-suppressive environment allows the virus to actively replicate, facilitating the production of lytic virus. In one example of this two-step scenario, the production of inflammatory mediators like tumor necrosis factor alpha (TNF-α), interleukin-2 (IL-2), and gamma interferon (IFN-γ) following allogeneic transplantation of kidneys in mice initiated IE gene expression [54]. However, full lytic reactivation of MCMV was only found in immune-deficient recipients [55]. Similarly, in human transplant patients, HCMV reactivation correlated with increased inflammatory responses [56, 57]. A second model focusing on latency and reactivation in the lung suggests that virus is reactivating frequently in immunocompetent hosts as shown by detectable levels of the immediate-early ie1/ie3 transcripts but that checkpoints exist which prevent full production of infectious virus following transcriptional activation [58–60]. Increased TNF-α levels were not enough to fully reactivate virus, although five-fold higher levels of IE transcripts were detected. Presumably memory T cells, specific for the IE proteins, lyse the reactivating cells before virus is produced [9, 58–61]. Multiple immune cells such as CD4+ and CD8+ T cells and other factors such as antibody and IFN-γ have also been shown to be important for maintaining latency [48, 62]. However, the immune cells and molecular factors that control viral latency and whether these cells and factors are the same between different tissues remains unclear.

In recent work, recurrent virus reactivation was accompanied by a concomitant increase in Treg frequency and suppressive functionality [63]. Thus, while Treg have been associated with viral reactivation, a causative role in control of latent CMV/MCMV has not been established. Here, we investigated the role of Treg in controlling latent MCMV infection. Strikingly, we found that Treg had opposing effects in distinct tissues harboring latent virus. In the spleen, Treg promoted viral persistence and suppressed local MCMV-specific effector T cell responses, while in the SG Treg were required to prevent viral reactivation/replication and IL-10 production by Foxp3- CD4+ T cells. These data show that Treg play divergent and tissue-specific roles in controlling viral reactivation from latency depending upon the type of T cells (effector or regulatory) they inhibit.

## Results

### Activated Treg are increased in the spleen during latent MCMV

**Fig 1:**
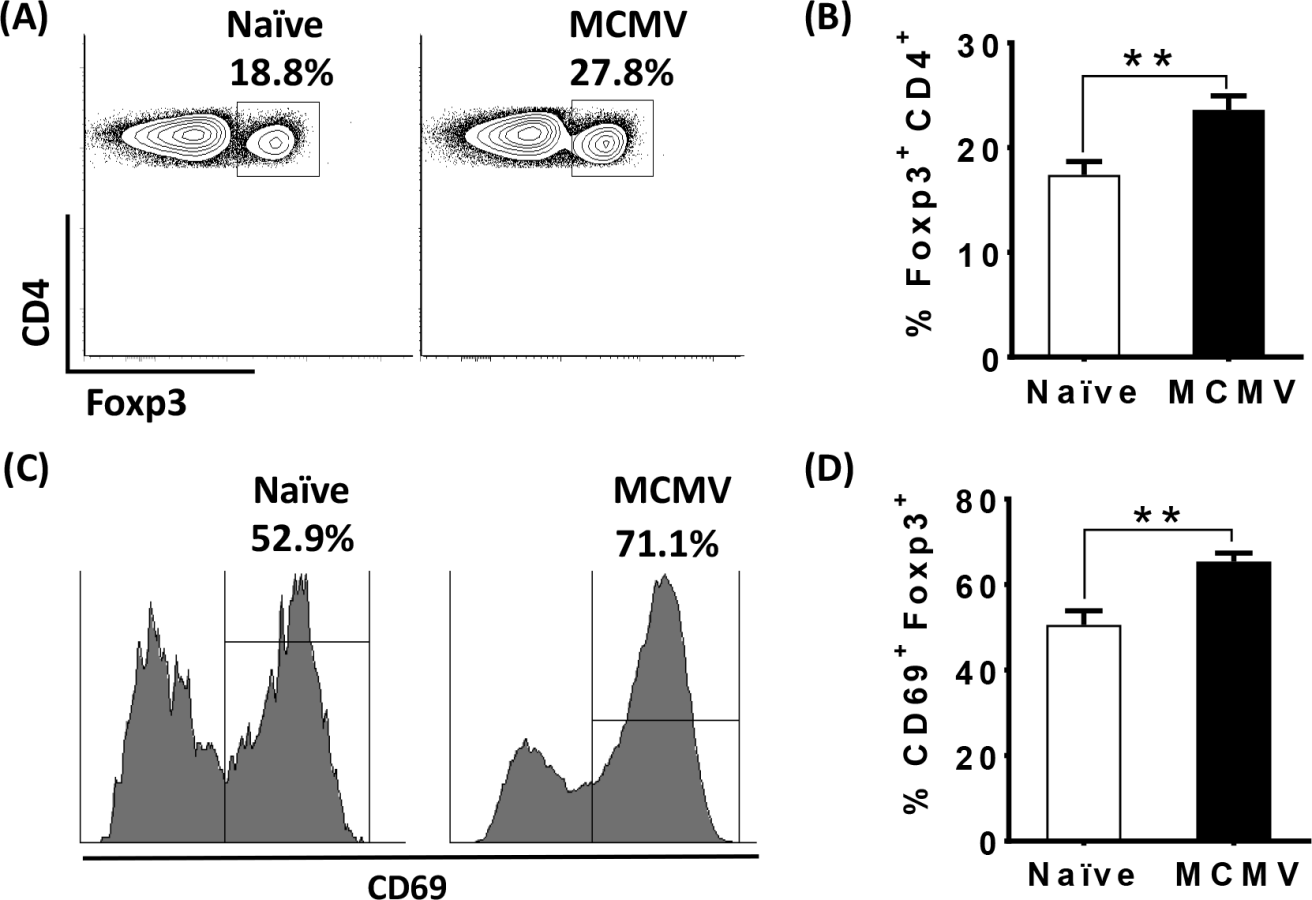
Activated Treg are increased in the spleen during latent MCMV. Splenocytes were isolated from naïve (9.5months old, white bars), or aged matched MCMV-latently infected mice (black bars, 8 months post- inoculation with1× 10^6^ pfu of MCMV). Cells were stained for CD4, CD69, and Foxp3 and analyzed by flow cytometry. Plots (A) and bar graph (B) show the frequency of Foxp3+ cells in total CD4+ cells from naïve and MCMV infected mice as described above (mean+SEM). Representative histograms (C) and bar graph (D) show the frequency of CD69+ on gated Foxp3+ cells from naïve and MCMV infected mice as described above (mean+SEM). Data is representative of two independent experiments. Naïve (N = 4), WT MCMV infected (N = 6). Statistical analysis (**p ≤ 0.01, Student’s *t*-test).

Foxp3+ regulatory T cells (Treg) can suppress effector T cells and promote MCMV replication in the spleen and salivary gland (SG) during acute MCMV infection [32]. However, there are no studies examining the role of Treg during latent MCMV infection. To investigate the role of regulatory T cells (Treg) during latent MCMV infection, we infected cohorts of C57BL/6 and Foxp3-DTR mice with MCMV and waited for 8 months. Notably, in the C57BL/6 background, low level virus replication continues in the SG and spleen for months longer than is observed in mice on a BALB/c background. Thus, it took 7 to 8 months following infection for the establishment of latency, as measured by the inability to detect replicating virus in multiple tissues, including spleen, lung, liver, pancreas, and SG (S1 Table). Next, we examined the levels and activation status of Treg in the spleen of latently infected mice. Interestingly, compared to uninfected mice of the same age, the frequency of Treg (Fig 1A and 1B), and in particular “effector” Treg (as assessed by CD69 expression) were significantly increased in MCMV infected mice (Fig 1C and 1D), although the total number of these cells was not different (S1 Fig). Thus, latent MCMV infection favors the preferential accrual of activated Treg in the spleen.

### Treg inhibit effector T cell responses and promote latent MCMV infection in the spleen

**Fig 2:**
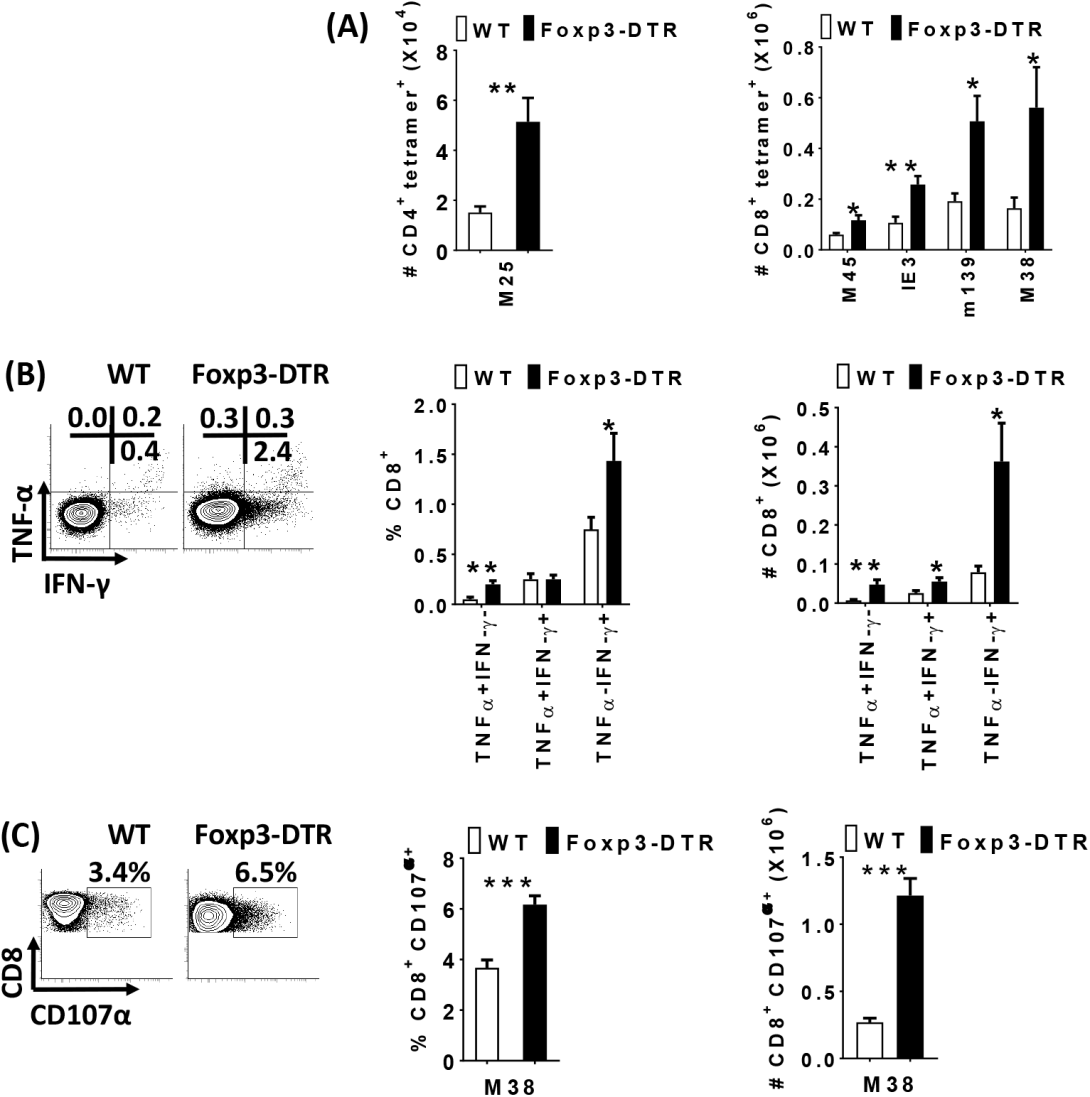
Treg inhibit effector T cell responses during latent MCMV infection in the spleen. 5–6 week old WT C57BL/6 (white bars) and Foxp3^**DTR**^ (black bars) mice were inoculated with1× 10^6^ pfu of MCMV. 8 months post-MCMV infection, both groups were injected with Diphtheria toxin (DT) on day 0, 3, 6 and sacrificed on day 7. Splenocytes from infected mice were stained with M25 (CD4 T cell) and M45, IE3, m139, M38 (CD8 T cell) tetramers day 7 (+DT) and analyzed with flow cytometry. Bar graphs (A) show the total number of M25- specific CD4 T cells and M45-, IE3-, m139- and M38-specific CD8 T cells from MCMV infected WT C57BL/6 (white bars, N = 6–7) and Foxp3^**DTR**^ (black bars, N = 6–8) mice (mean+SEM). Data are pooled from two independent experiments. Spleen cells (2 x 10^6^ cells/well) from MCMV infected mice day 7 (+DT) were *ex vivo* stimulated with M38 peptide (5 h at 37°C) and treated with Brefeldin A and were stained for CD8, and IFN-γ, TNF-α and analyzed by flow cytometry. Representative plots and bar graphs (B) show the average frequency and total number of CD8^+^ T lymphocytes producing IFN-γ and/or TNF-α (mean+SEM). WT C57BL/6 (N = 6), Foxp3^DTR^ (N = 6). Spleen cells from infected groups (+DT) were ex-vivo stimulated with MCMV-peptide (M38) for 5 hours during which CD107α antibodies were incubated with the cells. Representative plots and bar graphs (C) show the average frequency and total number of CD8+T lymphocytes expressing CD107α (mean+SEM).WT C57BL/6 (N = 11), Foxp3^DTR^(N = 10). Statistical analysis, *p ≤ 0.05, **p ≤ 0.01, ***p ≤ 0.001 (Student’s *t* test).

Next, we determined the role of Treg in latent MCMV infection using Foxp3-DTR mice, which allows the specific depletion of Treg using diphtheria toxin (DT) [64]. Before DT administration the levels of MCMV-specific T cells were not significantly different between WT and Foxp3-DTR mice (S2A Fig). After DT administration, the frequency and total numbers of Treg were substantially reduced relative to DT-treated controls (S2B Fig). As MCMV-specific T cells control viral replication, we determined the role of Treg in suppressing MCMV-specific T cell responses during latent infection. Treg depletion resulted in a significant increase in MCMV-specific CD8+ T and CD4+ T cells, relative to control animals (Fig 2A). MCMV-specific CD8+ T cells in Treg depleted mice expressed markers of differentiation (KLRG-1) and proliferation (Ki67) (S2C and S2D Fig). Functionally, the frequency of TNF-α or IFN-γ single producers and the numbers of single and double producers were significantly increased in Treg depleted mice (Fig 2B). Similarly, CD107α expressing MCMV-specific CD8+ T cells were significantly increased after Treg depletion (Fig 2C). Thus, Treg suppress effector T cell responses during latent MCMV infection.

**Fig 3:**
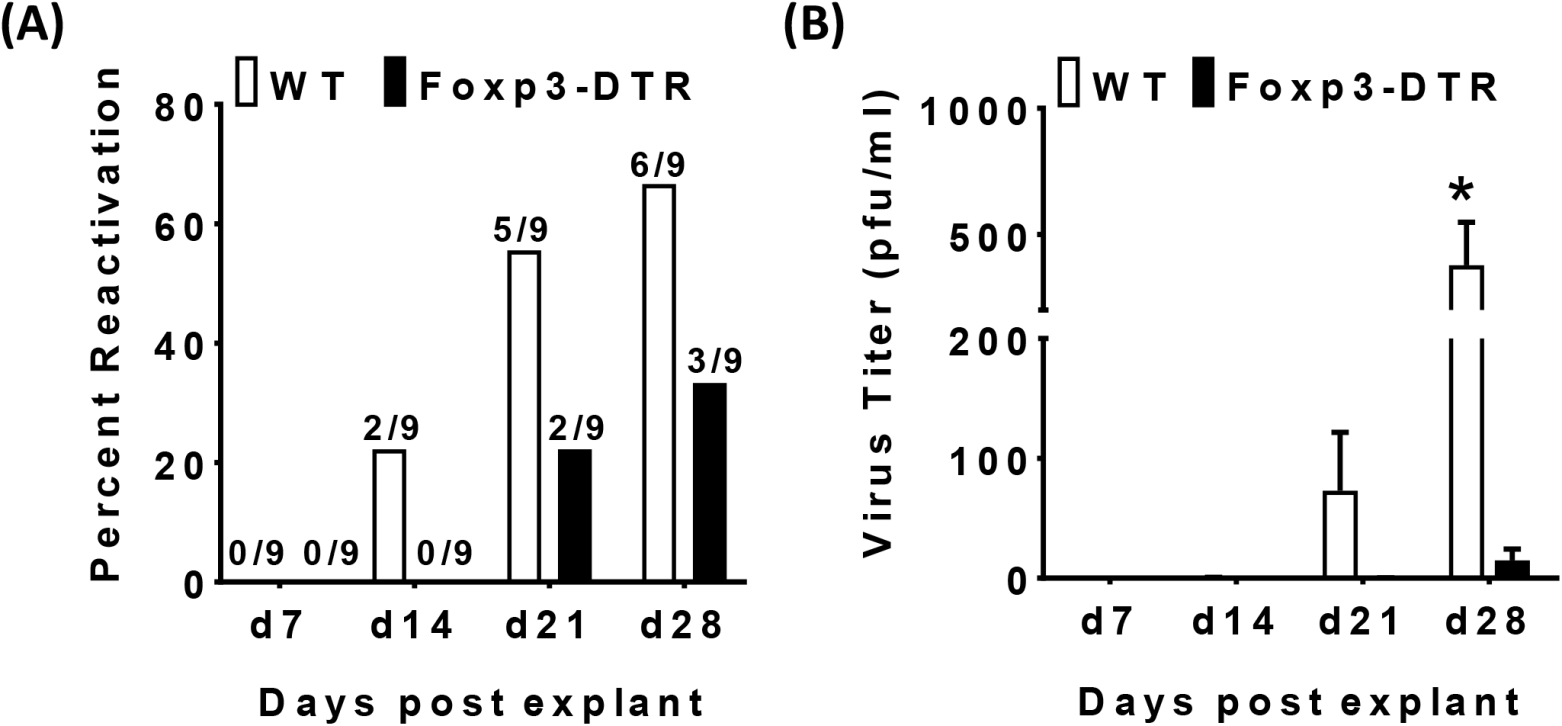
Treg promote latent MCMV infection in the spleen. 5–6 week old WT control and Foxp3^**DTR**^ mice were inoculated with 1× 10^6^pfu of MCMV (N = 9/group). 8 months post-MCMV infection, both groups were injected with Diphtheria toxin (DT) on day 0, 3, 6 and sacrificed on day 7. Spleens were analyzed for reactivation from latency by spleen explant assay as described in methods. Supernatants were assayed for infectious virus by plaque assay weekly. Bar graph (A) indicates the percentage of mice positive for virus reactivation from the spleens in either WT control (white bars) and Foxp3^**DTR**^ (black bars) mice, with the numbers of positive mice shown above the bars. Bar graph (B) represents average of virus titer of replicating virus in the supernatants of the spleen explant cultures of WT control (white bars) and Foxp3^**DTR**^ (black bars) MCMV infected mice day7 post Treg depletion (mean+SEM). Data include all of the mice in the experiment, including those mice with undetectable virus. A total of 1.5 ml of the explant cultures, representing ~37% of the total supernatant, was titered. Mice with undetectable amounts of virus were given a value of zero. Statistical analysis, (Student’s *t* test) *p ≤ 0.05.

Given the differences in effector T cell responses following Treg depletion, it was important to evaluate if loss of Treg would modulate the latent MCMV viral load. Seven days after initial DT treatment there was no actively replicating virus in the spleen in either controls or Foxp3-DTR mice as assessed by plaque assay (S2 Table). Using a spleen explant assay [65], we examined the impact of Treg on the latent viral pool. The spleen explant assay provides a functional assay to assess reactivation from latency, and thus, indirectly provide information on viral load levels since a lower latent viral load is expected to be less efficient at reactivation [65]. As detected in the explant assay, for the WT control mice, 6 out of 9 mice had evidence of viral reactivation by 28 days post explant culture, whereas MCMV reactivated only in 3 out of 9 Foxp3-DTR mice (Fig 3A). Further, titers of reactivating virus in WT mice were significantly higher in the first two weeks than those detected in the few Treg-depleted mice that had reactivated MCMV (Fig 3B). To further evaluate whether reduced virus reactivation from the spleen also correlated with reduced levels of viral DNA following Treg depletion, we performed qPCR on total splenic DNA to quantify viral genomes. Although there was variation from one experiment to the next, we saw a consistent decrease in viral genome levels in spleens from Foxp3-DTR mice relative to C57BL/6 controls across four independent experiments (S3 Fig, average percent decrease of 62.72% +/- 9.4; p<0.007). Thus, Treg are critical to maintain the reactivatable latent pool of MCMV in the spleen. However, the increase in overall spleen cellularity (and hence DNA content) following Treg depletion could also contribute to a decreased detection of viral load.

### Treg are required to prevent MCMV reactivation and MCMV-specific CD4+ T cells in the SG

**Fig 4:**
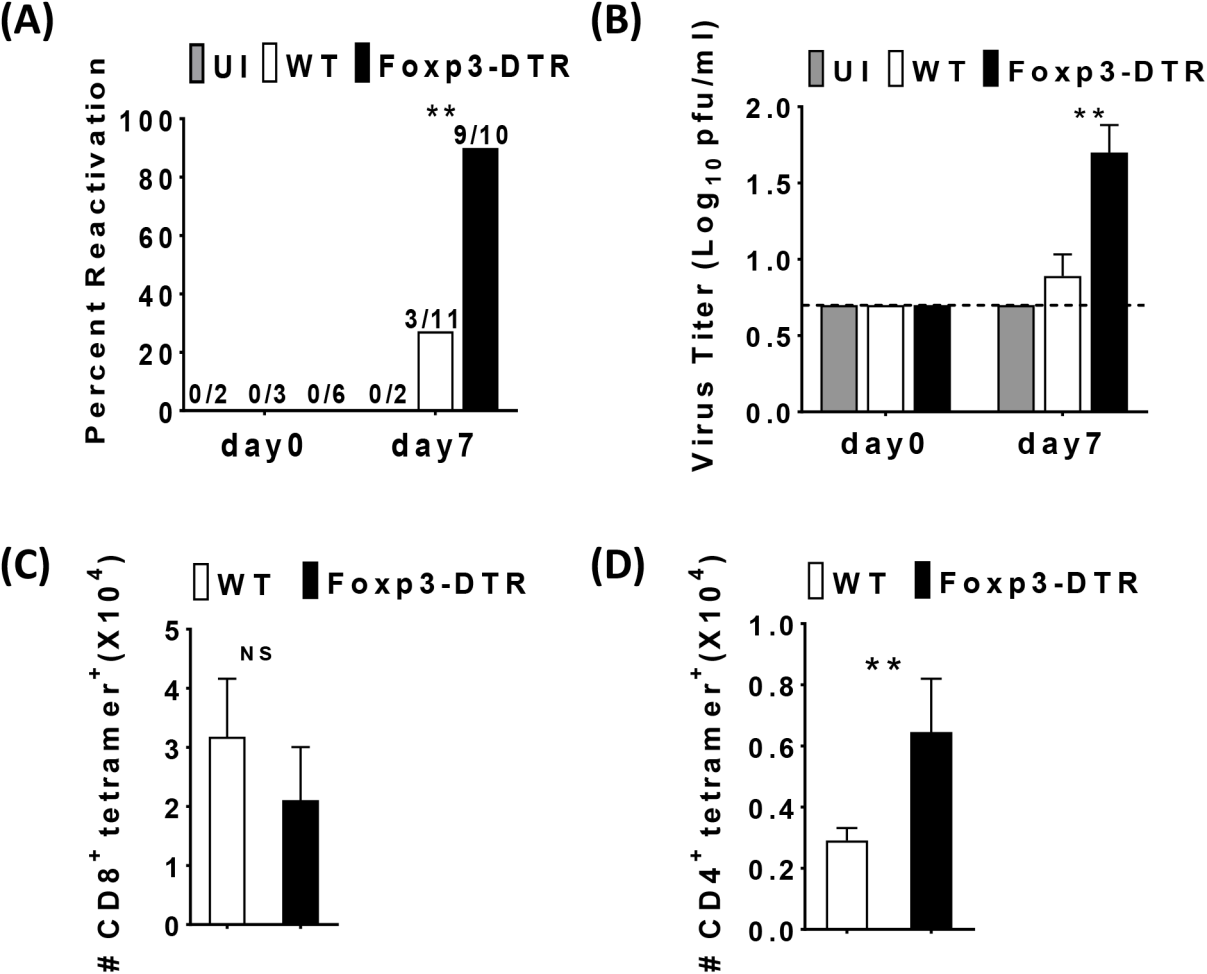
Treg are required to prevent MCMV reactivation and MCMV-specific CD4+ T cells in the SG. 5–6 week old WT C57BL/6 and Foxp3^**DTR**^ mice were inoculated with1× 10^6^ pfu of MCMV. 8 months post-MCMV infection, both groups were injected with Diphtheria toxin (DT) on day 0, 3, 6 and sacrificed on day 7. Bar graph (A) shows the percentage of WT C57BL/6 uninfected control (UI, N = 2 day 0, N = 2 day 7, gray bar), MCMV infected (N = 3 day 0, N = 11 day7 white bars) and Foxp3^**DTR**^ (N = 6 day 0, N = 10 day 7, black bars) mice positive for virus replication in the SGs before Treg depletion (indicated as day0) and day7 post Treg depletion with the numbers of positive mice in each group shown above the bars. (B) Bar graph shows the average viral titer of individual SGs of MCMV infected mice before Treg depletion (indicated as day0) and day7 post Treg depletion as described above (mean+SEM). The presence of replicating virus was detected by plaque assay. Samples with no detectable virus were assigned a titer of 0.7 log pfu/ml, the limit of detection for the plaque assay as indicated by the dashed line. Data is representative of five independent experiments. Statistical analysis, *p ≤ 0.05, **p ≤ 0.01 (Student’s *t* tests) comparing infected mice. Single cell suspensions were generated from the SGs of MCMV infected mice and were stained first with MCMV-tetramers, M38 (CD8), M25 (CD4) and then cells were surface stained for CD8, CD4, and analyzed with flow cytometry. Bar graph (C) shows the total number of M38-specific CD8 T cells in the SGs (mean+SEM). WT C57BL/6 (N = 4). Foxp3^DTR^ (N = 5). Bar graph (D) shows the total number of M25-specific CD4 T cells in the SGs (mean+SEM). WT C57BL/6 (N = 6). Foxp3^DTR^ (N = 8). Statistical analysis, *p ≤ 0.05, **p ≤ 0.01 (Student’s *t* test).

Given the role of Treg in promoting viral persistence in the spleen, we next determined the role of Treg in the control of latent MCMV infection in the SG. Similar to the spleen, latency in the SG was established by 8 months as evidenced by the lack of replicating virus prior to DT administration (Fig 4A). However, in stark contrast to the reduction in viral load in the spleen upon Treg depletion at day 7, Treg depleted mice had detectable replicating virus in the SG. While 3 out of 11 WT mice had a barely detectable viral reactivation, 9 out of 10 DT-treated Foxp3-DTR mice had a significant increase in viral reactivation and viral titers in the SG (Fig 4A and 4B). Indeed, in several experiments, virus was not detected in the WT SG whereas actively replicating virus was consistently detected in the SG of the Foxp3-DTR mice (Fig 4A and 4B). Additionally, reactivation occurred as early as day 4 after Treg depletion with evidence of increased, albeit low, viral titers in reactivating mice observed at that time (S4A and S4B Fig). Thus, in stark contrast to the spleen, Treg are critical to prevent lytic viral reactivation in the SG.

The SG provides a site for MCMV viral persistence and is a major site for accrual of tissue resident MCMV-specific memory CD8+ and CD4+ T cells [30, 66], although MCMV-specific CD8+ T cells are unable to drive viral clearance in this organ [22]. Given that Treg inhibited viral reactivation in the SG, we next investigated the SG T cell responses following Treg depletion in latently MCMV-infected mice. First, we established that DT administration efficiently depleted Treg in the SG (S5A Fig). In contrast to the spleen, we did not observe any change in the MCMV-specific CD8+ T cells (Fig 4C) or tissue resident MCMV-specific CD8+ T cells (CD103+CD69+) (S5B Fig) in Treg depleted mice. However, depletion of Treg resulted in an increase in the number of MCMV-specific CD4+ T cells in SG (Fig 4D). Thus, Treg suppress CD4+ but not CD8+ T cells in the SG during latent infection.

### Treg suppress CD4+ Foxp3- IL-10+ cells in the SG and IL-10 is critical for limiting viral load in the SG after Treg depletion

**Fig 5:**
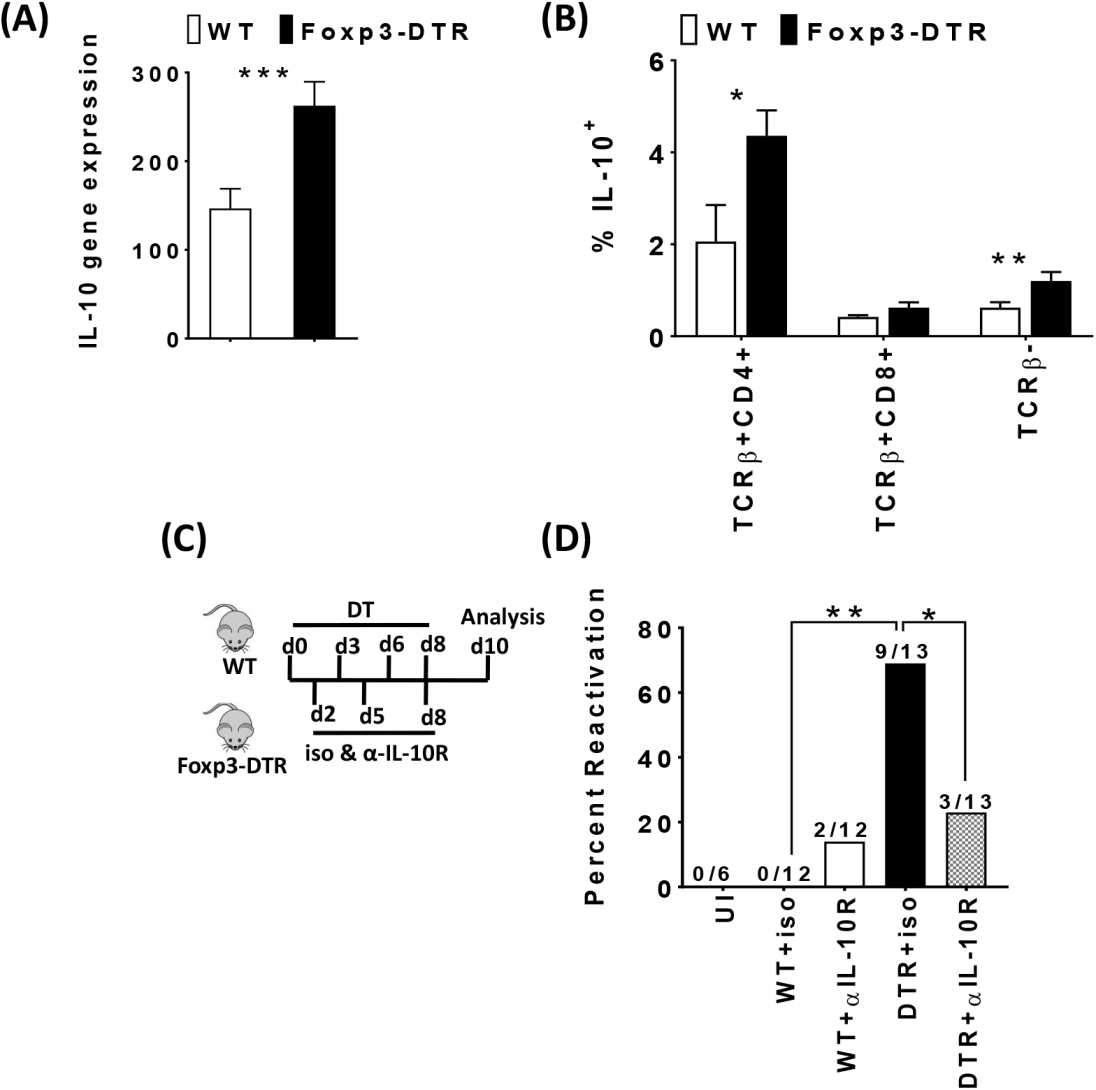
Treg suppress CD4+ Foxp3- IL-10+ cells in the SG and IL-10 is critical for limiting viral load in the SG after Treg depletion. 5–6 weeks old WT C57BL/6 (white bars) and Foxp3^DTR^ (black bars) mice were inoculated with 1× 10^6^ pfu of MCMV. 8 months post-MCMV infection, both groups were injected with Diphtheria toxin (DT) on day 0, 3, 6 and sacrificed on day 7. Bar graph (A) shows the average normalized IL-10 mRNA level (mean+SEM) in SG extracts of MCMV-infected mice (day7) post Treg depletion. WT C57BL/6 (N = 11). Foxp3^DTR^ (N = 10). Single cell suspensions were generated from the SGs of MCMV infected mice (day7 post Treg depletion). Cells were stained for TCRß, CD4, CD8, Foxp3 and IL-10 following stimulation with or without PMA and ionomycin for 5 hours, in the presence of brefeldinA. Bar graph (B) shows the average of frequency of IL-10+ in Foxp3- CD4+ TCRβ+ and CD8+ TCRβ+ cells and TCR-β− cells (mean+SEM) in the SG. WT C57BL/6 (N = 5). Foxp3^DTR^ (N = 5). Statistical analysis, *p ≤ 0.05, **p ≤ 0.01 (Student’s *t* test). (C) Schematic representation of diphtheria toxin and IL-10 neutralization treatment. Briefly, 5–6 week old WT C57BL/6 (N = 24) and Foxp3^**DTR**^ (N = 26) mice were inoculated with 1× 10^6^ plaque-forming unit (pfu, p.i.) of MCMV. 8–12 months post-MCMV infection, both groups were injected with DT on day 0, 3, 6, 8 and sacrificed on day 10. Groups were split and were treated with 500μg of either isotype control antibody or with anti–IL-10R neutralizing antibody on day 2, 5, 8 and were sacrificed on day10. Bar graph (D) indicates the percentage of mice positive for virus reactivation in the SGs (day 10) post Treg depletion and IL-10R neutralization with the numbers of mice in each group shown above the bars. WT C57BL/6 uninfected (UI, N = 6), WT C57BL/6 MCMV infected (N = 24) and Foxp3^**DTR**^ (N = 26). The presence of replicating virus was detected by plaque assay (data combined from two independent experiments). Statistics for percentage of mice with virus reactivation which was made using Fisher’s exact test (*P ≥ 0.*05*, ** P≥ 0.*01*) comparing all groups.

Prior work showed that IL-10 is critical for promoting viral replication in the SG [27, 28]. Indeed, we found that depletion of Treg drove a significant increase in SG IL-10 mRNA levels (Fig 5A). Given the increase in CD4+ T cell responses in the SG, we next examined the potential cellular sources of IL-10. Importantly, CD4+Foxp3-cells were the predominant source for IL-10 production compared to CD8+ T cells and non–T cells (Fig 5B). Combined, these data show that Treg suppress a population of CD4+ Foxp3- IL-10+ cells, consistent with a potential role of these cells in MCMV replication in the SG. As the effects of Treg depletion on viral reactivation/latency were quite different in the SG compared to the spleen, we examined IL-10 levels in the spleen as reduced IL-10 could potentially explain the different viral loads. Similar to the SG, IL-10-producing Foxp3- CD4+ T cells were increased in the spleen (S6A Fig). However, unlike the SG, total IL-10 mRNA was not increased in the spleen, suggesting that the total amount of IL-10 in the spleen is not substantially increased (S6B Fig).

To further investigate the role of IL-10 in the SG after Treg depletion, we blocked IL-10R signaling by administration of a blocking IL-10R antibody with and without Treg depletion using Foxp3-DTR mice (Fig 5C). As expected, Treg depletion was again accompanied by an increase in the number of mice harboring reactivating virus as measured by plaque assay (Fig 5D). Strikingly, neutralization of IL-10 during Treg depletion significantly reduced the total number of mice with MCMV reactivation (9/13 to 3/13) (Fig 5D). Thus, our data show that Treg limit IL-10 production in the SG and that IL-10 is essential for viral reactivation/replication in the SG after Treg depletion.

## Discussion

Investigating the role of regulatory T cells in latent MCMV has been hampered by the lack of appropriate tools. Our ability to assess the role of Treg in latent MCMV infection was greatly facilitated by two essential tools. First, Foxp3-DTR mice, which express diphtheria toxin receptor under the Foxp3 promoter allowed for depletion of Treg upon DT administration[64]. Second, the use of a sensitive tissue explant assay and qPCR allowed us to assess the reactivatable latent viral pool in mice with Treg depletion [65, 67]. Herein, we found that depletion of Treg during latent MCMV infection had profound consequences. In the spleen, Treg restrained MCMV-specific CD4+ and CD8+ T cells and promoted the latent viral pool. In stark contrast, in the SG, Treg were essential to limit viral reactivation because they prevented the emergence of IL-10- secreting Foxp3- CD4+ T cells. Thus, we demonstrate unique tissue-specific functions of Treg in control of latent MCMV infection.

During latent MCMV infection, we found a significant increase in activated Treg in the spleen. We speculate that the increase in Treg maybe a direct consequence of the chronic stimulation due to the periodic low-level viral reactivation during latency. In agreement, in other chronic infections like Leishmaniasis, hepatitis C virus (HCV), hepatitis B virus (HBV) and human immunodeficiency virus (HIV), Treg frequency was substantially increased in the spleen and other tissues [68–70]. Interestingly, human herpes virus 6 (HHV-6) infection, another herpes virus closely related to HCMV, induces virus-specific CD4+ and CD8+ regulatory T cells [71]. Thus, chronic infections appear to promote their own persistence by driving Treg accrual.

Strikingly, Treg depletion resulted in markedly reduced latent virus in the spleen as measured by two assays. Some mice had undetectable viral DNA levels in the spleen, suggesting that viral load had been reduced to levels that were below the limit of detection in our assay. We acknowledge that the increase in spleen size and cellularity could contribute to the observed reduction in latent viral load. However, in Treg-depleted mice where viral DNA load was undetectable, the spleen size and cellularity was similar to mice with detectable levels of viral DNA. Nonetheless, future experiments will investigate this in more detail, examining the number of cells harboring latent virus and the levels of virus within such cells. The spleen explant assay allowed us to assess one aspect of latent MCMV infection, the ability to reactivate from latency as a measure of whether reactivatable latent viral loads in the spleen were affected by Treg depletion. In our hands, if virus is replicating in the spleen at the time of isolation, this is detected by seven days post explants [65, 72]. However, if virus is latent in the tissue at the time of isolation, it takes longer for the virus to be detected in the explant assay, usually by day 14, and then rapidly spreads and replicates within the culture. Although this assay is not quantitative per se, we recently demonstrated that a mutant virus was unable to reactivate from the spleen by explant culture and had reduced levels of viral DNA [65], (Cardin, manuscript in preparation). Here, our results show that the Foxp3-DTR mice were less efficient at reactivation in this assay, suggesting lower levels of latent virus in the spleen. Importantly, we found that both the number of Foxp3-DTR mice with reactivating virus and also the levels of virus replication per mouse in the spleen explant cultures were reduced. Decreased reactivation from the spleen was highly reproducible between studies, and indeed, qPCR analysis of viral DNA in the spleen could indicate lower levels of latent virus in mice depleted of Treg, with the caveat of spleen size as mentioned earlier. Alternatively, we cannot rule out that the increased numbers of virus-specific T cells in the Treg-depleted spleens are functional after placement into the explant culture and thus elimination of latently-infected cells could also occur *in vitro*; however, these T cells likely have a very limited survival *in vitro*. It is important to note that we were unable to carry out long-term Treg depletion (greater than 14 days), because of the rampant and lethal autoimmunity that ensues with long-term Treg depletion in these mice [64]. Further, the impact of Treg depletion on latent viral genomes likely only impacted those genomes that underwent a reactivation event and became detectable by the immune system due to viral antigen expression. Given that Treg depletion was accompanied by a substantial increase in cytotoxic MCMV-specific CD8+ T cells in the spleen, it is likely that Treg depletion led to the reactivation of some viral genomes, which was likely quickly quelled in the spleen by an MCMV-specific CD8+ T cell response, possibly even before lytic virus was produced [49]. Additional studies are needed to address this further.

One important question raised by our study is the mechanism(s) by which Treg suppress CD8+ T cell responses to MCMV. Prior *in vitro* work suggested that Treg utilize TGF-β to suppress MCMV- specific T cell responses [31], although CTLA-4 and IL-10 may also have a regulatory role [73, 74]. In a chronic Friend virus (FV) infection model, FV-specific CD8+ T cells were also partially restrained by Treg, although the mechanism(s) appear to be independent of PD-1 and Tim-3 [75]. Interestingly, our previously published data show that Treg from aged mice have significantly increased levels of IL-35p19, suggesting that IL-35 may contribute to suppression of MCMV-specific CD8+ T cell responses [76]. However, a recent paper showed that IL-35 produced by NK cells during acute MCMV infection promotes, rather than inhibits IL-10 production [77]. Nonetheless, further studies are required to determine the mechanism by which Treg restrain MCMV-specific CD8+ T cell effector functions and thus promote latent infections.

It was very surprising that, while the depletion of Treg resulted in reduction in viral load in the spleen, it was accompanied by an augmented reactivation and/or replication of the latent viral pool in the SG. We envision three major potential mechanisms that may explain these results. First, it is possible that under chronic infection such as that observed in MCMV infected SG, Treg could lose immunosuppressive abilities and acquire the phenotype of a more pathogenic or anti-viral Treg. There is precedence for Treg metamorphosis in tumor models and under chronic inflammatory conditions [78–81]. This makes teleological sense as chronic inflammation may favor Treg with more effector function to replace exhausted effector T cells. However, we looked for Treg expression of pathogenic markers (i.e. T-bet, RORγt) on SG Treg and failed to find expression of these markers. Second, it is possible that the kinetics of latency establishment between the two tissues explains the differential effects on latent virus (clearance & reactivation) in the two tissues. For example, because the virus established latency in the spleen after three months but was latent in the SG after 7 months, perhaps clearance was easier to achieve in the spleen due to a lower level of latent viral load. However, we performed several experiments examining Treg depletion 2–5 months after primary infection and found that, similar to 8 months after infection, that loss of Treg drove significant decrease in MCMV replication in the spleen (S7 Fig). Thus, it is likely that the tissue microenvironment rather than the timing controls the tissue-specific role of Treg. Third, it is possible that Treg maintain their suppressive capacity and instead of their “classical” role of inhibiting pro-inflammatory T cells, it is possible that they similarly control anti-inflammatory T cells. If so, the result of suppressing anti-inflammatory cells would be the promotion of an immune-suppressive environment which is permissive for viral reactivation/replication. Our data are most consistent with this latter possibility. In this regard, CD4+ T cell production of IL-10 was increased in both the spleen and SG after Treg depletion. One obvious question is why didn’t this increase in IL-10 drive viral reactivation in the spleen? One likely explanation is that, in the spleen, where CD8+ T cells are critical to prevent lytic virus production, their levels of IL-10R are significantly decreased, making them insensitive to IL-10 [82].

Our data also show that Treg depletion drives viral reactivation in the SG and implicates IL-10 in the process. However, current data in the literature suggest that IL-10 contributes more to viral replication than outright reactivation [83]. Indeed, prior work has shown that the specific production of IL-10 from Foxp3- CD4+ cells attenuates acute antiviral immune-responses and leads to persistent viral replication in the SG [27]. Thus, while our data clearly show that inhibition of IL-10R signaling restored viral control, more work is required to conclusively determine whether IL-10 promotes outright reactivation or promotes viral replication. However, in our study, we initiated IL-10R antibody treatment following the initiation of Treg depletion, thus, if virus had started to reactivate, it could have been effectively controlled. The effect of IL-10 on viral control could be direct or indirect. IL-10 can directly affect CD4+ T cell production of effector cytokines like IFN-γ [84]. Indeed, IFN-γ is indispensable for the control of viral load in the SG [25, 85]. However, there was no diminution in IFN-γ production upon Treg depletion, instead IFN-γ production was actually increased (S8 Fig). Alternatively, IL-10 can indirectly compromise CD4+ T cell responses in the SG by interfering with the responsiveness of APC to IFN-γ. For example, it is well known that IL-10 inhibits the effect of IFN-γ by interfering with IFN-γinduced genes, preventing the phosphorylation of STAT-1 molecules and activation of monocytes [86]. Thus, while our data clearly show a role for IL-10, more work is required to determine the cellular targets of IL-10 that regulate reactivation/replication. Notably, the cellular site(s) of MCMV latency in host tissues is a long debated issue. Like HCMV, MCMV can establish a latent infection in cells of the myeloid lineage and these cells are able to respond to IL-10. However, other, non-hematopoietic targets, like endothelial cells cannot be excluded.

This divergent and tissue-specific role in controlling MCMV viral latency by Treg raises the question as to whether Treg manipulation is a useful therapy in latent HCMV infection. In HCMV, manipulating Treg is viewed as a promising therapeutic approach to control latent viral reactivation in immune-compromised hosts like organ transplant patients. In a prior study, the use of immune-suppressive drugs like daclizumab (anti-CD25), steroids and calcineurin inhibitors in CMV sero-positive renal transplant patients, led to reduction of Treg levels that correlated with enhanced levels of CMV-specific effector T cells, suggesting that modulation of Treg favors maintenance of CMV-specific immunity [87]. However, Treg depletion may also promote viral reactivation in sites such as SG that are not assessed in treated patients. Thus, manipulating Treg could be a double-edged sword. Treg in CMV infection might exert different functional activity depending on their localization within the infected host and the T-cell responses that they regulate. Our data, suggesting that Treg could regulate another suppressor CD4+Foxp3-IL-10+ cells in the SG and limit viral reactivation opposed to their conventional role in regulating effector T cells as observed in the spleen, is a novel concept. It is unclear whether the mechanisms employed by Treg to inhibit effector T cell responses vs. other regulatory cell populations are distinct. Understanding such mechanisms might be crucial however to enhance the suppressive effects of Treg on other immune suppressive cell populations in some instances (chronic infections) or block the suppressive effect of Treg on others (i.e. autoimmunity).

## Methods

### Cells and virus

NIH 3T3 cells (ATCC CRL1658) were grown in Dulbecco’s modified Eagle’s medium (DMEM, Media tech, Herndon, VA) supplemented with 10% fetal bovine serum (FBS, Hyclone, Logan, UT), 7.5% Sodium Bicarbonate, 4 mM HEPES, 2 mM L-glutamine, and gentamicin in a humidified 5% CO_2_ incubator at 37°C. Parent stocks of the wild type MCMV K181 (originally a kind gift from Dr. Ed Mocarski, Stanford University) were prepared in NIH 3T3 cells from a SG-derived virus stock as previously described [65]. Virus titers were determined by plaque assay on NIH 3T3 cells. All virus stocks were stored at -70°C and re-titered before use in experiments.

### Mice and infection

Young C57BL/6 mice were purchased from Taconic Farms (Germantown, NY).

Foxp3-IRES-DTR-GFP knock-in C57BL/6 mice were a generous gift from Dr. A. Rudensky. (Rockefeller University, NY). For depletion of Foxp3 Treg cells, 1μg DT was injected at day 0. Followed by 0.25μg DT on day 3, 6. Mice were sacrificed on day 7. For Treg depletion and IL-10R neutralization, mice were injected with 1μg DT at day 0, followed by 0.25μg DT on day 3, 6 and 8. In addition, mice received antiIL-10R blocking antibody (Clone: 1B1.3A BioXcell) or ratIgG1 isotype control (Clone: HRPN BioXcell) 500μg on day 2, 5, 8 and 250μg at day 8. Mice were sacrificed on day 10. All mice were injected by intraperitoneal route (i.p.).

For MCMV infection, five to six week-old mice were infected with MCMV K181 strain, via i.p. inoculation with 1 × 10^6^ PFU. Mice were sacrificed upon establishment of latent MCMV at various times (> 6–7 months post infection in C57Bl/6 mice). Mice were maintained under specific-pathogen-free conditions at Cincinnati Children's Hospital Medical Center.

### Ethics statement

All animal protocols were reviewed and approved by the Institutional Animal Care and Use Committee at the Cincinnati Children’s Hospital Research Foundation (CCHRF) under IACUC2016-0087 (1D03023).

The care and use of laboratory animals at CCHMC is in accordance with the principles and standards set forth in their Principles for Use of Animals (NIH Guide for grants and Contracts), the Guide for the care and Use of Laboratory Animals (Department of Health, Education, and Welfare DHEW, Public Health Service PHS, National Institutes of Health NIH Publ. 8th edition, Rev.2011). The provisions of the Animal Welfare Acts (P.L. 89–544 and its amendments), and other applicable laws and regulations.

### Virological methods

For plaque assays, dilutions of virus stocks, 10% (w/v) mouse tissue sonicates, and sonicated leukocyte cell suspensions were adsorbed onto 70% confluent NIH 3T3 monolayers for one hour at 37°C, and then overlaid with 1:1 carboxymethyl cellulose (CMC): 2X modified Eagle’s medium as previously described[85]. At 6 days, the overlay was removed and the cells were fixed with methanol and stained with Giemsa to determine the number of plaques. For measurement of tissue virus replication following mouse infection, tissues were placed in pre-weighed tubes or were homogenized in media to prepare cell suspensions, followed by sonication on ice to disrupt cells and release free virus. Tissue homogenates were titered by plaque assay similar to virus stocks as described above.

### Reactivation assays

Explant reactivation assays of tissues from latently infected mice were established as previously described [45, 65]. After infection when virus was no longer replicating and establishment of latency (> 6–7 months post infection in C57Bl/6 mice) and following DT treatment, the SGs and spleens were collected. In some experiments, other tissues such as lungs and liver were also collected for plaque assays. The SGs were sonicated and titered to detect persistent replicating virus as the SG is a major site of viral persistence. In some experiments, to analyze populations of infiltrating leukocytes in the SGs, the SGs were first homogenized to prepare a cell suspension, an aliquot was removed for sonication and plaque assay analysis, and the remaining sample was treated as described below under ‘cell isolation’. For the explant reactivation assays, the spleens were minced and primary spleen cultures were established as previously described [45].In some studies, SGs and lungs were also analyzed by explant reactivation assay. Briefly, the spleen explant assay was established by dividing the spleens into three parts, with each part placed into a well of a 6-well tissue culture plate containing 5 ml of media. The cultures were followed for up to 6 weeks and culture media from each well were collected weekly, sonicated and titered in plaque assays to detect the presence of reactivating or replicating virus. Plaques detected in any wells were counted as a reactivation event.

### PCR analysis

Tissues were isolated from infected and uninfected mice using dissection tools pre-treated with DNA Away (Molecular BioProducts). DNA was isolated from cell suspensions following tissue homogenization using QIAamp DNA Mini Kit (Qiagen #51306) according to manufacturer’s protocol. Quantitative PCR was performed as previously described[88] using the following primers: E1 forward primer 5’-TCGCCCATCGTTTCGAGA-3’; E1 reverse primer 5’-TCTCGTAGGTCCACTGACGGA-3’ to yield an amplified 106bp product. The TAMRA Taqman E1 probe was purchased from Applied Biosystems.

### Cell isolation

Spleens were harvested and crushed through 100-μm filters (BD Falcon) to generate single-cell suspensions. For the SG, tissue was digested with collagenase (4) or with liberase in media (RPMI 1640 containing 0.5 mg/ml digestion enzyme (Sigma), 5mM CaCl2, 0.2 mg/ml of DNase I (Sigma) and 5% FBS) incubated for 45minutes at 37°C, followed by a two-step discontinuous Percoll gradient (Little Chalfont, UK). Gradient samples were centrifuged at 25000rpm, room temperature, for 25 min with the brake off. The lymphocytes were harvested at the interface between 30% and 70% Percoll layers.

### Flow cytometry

A total of 1–2 million single-cell suspensions from either spleen or SG surface stained with a combination of the following tetramers and antibodies:

M45 –HGIRNASFI, m139 –TVYGFCLL, M38 –SSPPMFRV, IE3 –RALEYKNL andM25 –NHLYETPISATAMVI. Tetramers were all synthesized by the NIH tetramer core facility. Cells were then incubated with Fc block and surface stained with Surface Abs: αCD4, αCD8α, αTCRβ (BD Biosciences, San Diego, CA, USA), αCD44, αKLRG1, αCD127, αCD69, αCD103, αCX3CR1, αCD16/32 and αLy6C (eBioscience, San Diego, CA, USA). For CD8 T cell intracellular staining, cells were stained in media and fixed with 2%Methanol free formaldehyde (MF FA) for 1 hour and then intracellularly stained with αKi67 (eBioscience) using eBio permeabilization buffer. For CD8 T cell peptide stimulation using M38, M139 at 2μg/ml (a gift from Dr. Edith Janssen, Cincinnati Children's Hospital Medical Center, Cincinnati, OH) cells were stimulated for 5 h in the presence of anti-CD107a antibody and in the presence of brefeldinA for the final 4 h. Cells were then fixed with 2%MF FA for 1 hour and then in 0.05% saponin. Cells were stained for IFN-γ and TNF-α production (all from eBioscience). For CD4 T cells, cells were stimulated with 50ng/ml PMA and 1μg/ml ionomycin for 5 hours, in the presence of brefeldinA for the final 4 h and fixed with 2%MF FA for 1 hour followed by intracellular staining for IL-10, IFN-γ, TNF-α (all from eBioscience) and Foxp3 (eBioscience) staining was done according to eBioscience Foxp3 staining kit and protocol. Data were acquired on an LSRII flow cytometer (BD Biosciences) and analyzed using FACSDiva software (BD Biosciences).

### RT-PCR

Samples were homogenized and total cellular RNA was extracted and quantified. DNase-treated RNA was then used to synthesize cDNA. The primer sequences used for detection of IL-10 were: 5′- GCTCTTACTGACTGGCATGAG -3′ and 5′- CGCAGCTCTAGGAGCATGTG -3′.

The primer sequences used for detection of β-actin as an internal control were 5′-GGCCCAGAGCAAGAGAGGTA-3′ and 5′-GGTTGGCCTTAGGTTTCAGG-3′.

Quantitative real-time PCR was performed with Roche LightCycler 480 SYBRGreen 1 Master Mix using the Roche LightCycler 480 II instrument (Roche Diagnostics).

### Statistical analysis

Data were analyzed using GraphPad Prism software and Excel software. Statistical analysis was performed using either a Student’s *t*-test or a Fisher’s exact test in different experiments. A p-value of <0.05 was considered significant.

## Supporting information

S1 Table Establishment of latent MCMV infection. Table shows the number of mice with positive MCMV titers (replicating virus) in the spleen, lung, liver, pancreas and salivary gland within the three groups: Naïve, WT control and Foxp3^DTR^, 8 months post MCMV infection. Titers were quantified via plaque assay before Treg depletion, indicated here as Day0. 0/number of mice in each group indicates absence of actively replicating virus and confirms the establishment of latency in all tissues.
(PDF)

S2 Table Absence of actively replicating virus in the spleen in controls and Foxp3-DTR mice 7 days post Treg depletion. Table shows the number of mice with positive MCMV titers (replicating virus) in the spleen within the two groups: WT control and Foxp3DTR, 8 months post MCMV infection. Titers were quantified by plaque assay 7 days after Treg depletion, indicated here as Day7. 0/number of mice in each group indicates absence of actively replicating virus and confirms the establishment of latency. (N = 9/group).
(PDF)

S1 Fig Treg in the spleen during latent MCMV. Splenocytes were isolated from naïve (9.5months old), or aged matched MCMV-latently infected mice (8months p.i.). Cells were stained for CD4 and Foxp3 and analyzed by flow cytometry. Graph shows the number of Foxp3+ cells in total CD4+ cells. Naïve (N = 4), WT MCMV infected (N = 6).
(PDF)

S2 Fig Highly activated, proliferating MCMV-specific CD8+ T cells in the spleen post Treg depletion. 5–6 week old WT C57BL/6 and Foxp3^DTR^ mice were inoculated with 1× 10^6^ pfu of MCMV. 8 months post-MCMV infection, splenocytes were isolated from infected mice. Cells were stained for IE3, m139, M38 (CD8 T cell) tetramers day 0 (-DT) and analyzed with flow cytometry. A) Graph shows the total number of IE3-, m139- and M38-specific CD8 T from WT C57BL/6 (white bars, N = 3) and Foxp3^DTR^ (black bars, N = 6) mice. 5–6 week old C57BL/6 and Foxp3^DTR^mice were inoculated with1× 10^6^ pfu of MCMV. 8 months post-MCMV infection, both groups were injected with Diphtheria toxin (DT) on day 0, 3, 6 and sacrificed on day 7. Spleen cells were analyzed by flow cytometry. B) Bar graphs show the average frequency and absolute number of CD4+ Foxp3+Treg in the spleen (mean+SEM). WT C57BL/6 (N = 11). Foxp3^DTR^ (N = 10). Spleen cells isolated from the two groups were stained with MCMV CD8-specific tetramers and then surface stained for expression of KLRG-1 and CD127 and intra-cellular expression of Ki67. C) Bar graph shows the total numbers of effector subpopulations within gated m139-specific CD8 T cells (mean+SEM). WT C57BL/6 (N = 6), Foxp3^DTR^ (N = 6). D) Bar graphs show the frequency and absolute number of Ki67+ cells within M45-, IE3-, m139- and M38-specific CD8 T (mean+SEM). WT C57BL/6 (N = 6), Foxp3^DTR^(N = 6). Statistical analysis, *p ≤ 0.*05*, **p ≤ 0.*01*, ***p ≤ 0.*001 (*Student’s *t-test)*.
(PDF)

S3 Fig MCMV viral load in the spleen. Genomic DNA was isolated from the spleens of WT C57BL/6 and DTR mice at day 7 post treg depletion. MCMV E1 was detected by quantitative PCR, and data expressed as genome copy number per 100 ng genomic DNA as described in Materials and Methods. Results are pooled from 4 independent experiments (WT, N = 20 and DTR, N = 22) and show the mean+SEM. The average reduction in viral load in DTR mice in 4 independent experiments was 62.7% ± 9.4 (one sample t-test p<0.007).
(PDF)

S4 Fig Early MCMV viral reactivation post Treg depletion in the SG. 5–6 week old WT C57BL/6 (white) and Foxp3^DTR^ (black) mice were inoculated with 1× 10^6^ pfu of MCMV (N = 8/group). 9 months post-MCMV infection, both groups were injected with Diphtheria toxin (DT) on day 0, 3 and sacrificed on day4. A) Bar graph shows the percentage of mice positive for virus reactivation of naïve (UI) and MCMV infected mice in the SGs day4 post Treg depletion with the numbers of mice in each group shown above the bars. The presence of replicating virus was detected by plaque assay. B) Bar graph shows viral titers of individually homogenized SGs of naïve and MCMV infected mice Day4 post Treg depletion (mean+SEM).
(PDF)

S5 Fig Efficiency of Treg depletion in the SG. 5–6 weeks old WT C57BL/6 and Foxp3^DTR^ mice were inoculated with1× 10^6^ pfu of MCMV. 8 months post-MCMV infection, both groups were injected with Diphtheria toxin (DT) on day 0, 3, 6 and sacrificed on day 7. A single cell suspension from the SGs of all MCMV infected mice was analyzed by flow cytometry. A) Bar graphs show the average frequency and absolute number of CD4+ Foxp3+ Treg in the SG upon DT administration (mean+SEM). WT C57BL/6 (N = 7) Foxp3^DTR^ (N = 8). B) Bar graph shows the average frequency of tissue resident memory (CD103+CD69+) cells in the SG within the total M38-specific CD8 T cells (mean+SEM). C57BL/6 (N = 4). Foxp3^DTR^ (N = 5). Statistical analysis, *p ≤ 0.05, **p ≤ 0.01, ***p ≤ 0.001 (Student’s t-test).
(PDF)

S6 Fig Treg suppress CD4+ Foxp3- IL-10+ cells in the spleen. 5–6 weeks old WT C57BL/6 (white bars) and Foxp3^DTR^ (black bars) mice were inoculated with 1× 10^6^ pfu of MCMV. 8 months post-MCMV infection, both groups were injected with Diphtheria toxin (DT) on day 0, 3, 6 and sacrificed on day 7. A) Single cell suspensions were generated from the spleen of MCMV infected WT C57BL/6 (N = 11) and Foxp3^DTR^ (N = 10) mice (day7) post Treg depletion. Cells were stained for TCRß, CD4, CD8, Foxp3 and IL-10 following stimulation with or without PMA and ionomycin for 5 hours, in the presence of brefeldinA in the final 4hrs. Bar graph shows the average of frequency of IL-10+ in Foxp3- CD4+ TCRβ+ and CD8+ TCRβ+ cells and TCR-β− cells (mean+SEM). B) Graph shows the average normalized IL-10 mRNA level in spleen of MCMV-infected WT C57BL/6 (N = 10) and Foxp3^DTR^ (N = 9) mice (day7) post Treg depletion (mean+SEM). Statistical analysis, *p ≤ 0.05, **p ≤ 0.01 (Student’s *t* test).
(PDF)

S7 Fig Treg promote MCMV replication in the spleen. 5–6 week old WT C57BL/6 (white) and Foxp3^DTR^ (black) mice were inoculated with 1× 10^6^ pfu of MCMV (N = 8/group). 5 months post-MCMV infection, both groups were injected with Diphtheria toxin (DT) on day 0, 3, 6, 9,12 and sacrificed on day 14. A) Bar graph shows the percentage of mice positive for virus replication in the spleen day14 quantified by plaque assay post Treg depletion with the numbers of mice in each group shown above the bars. Viral titers were 18.6 pfu/ml +/- 15.5 in WT C57BL/6 mice and 2.4 pfu/ml +/- 2.24 in FoxP3-DTR mice; p = 0.31. B) Genomic DNA was isolated from the spleens of WT C57BL/6 and DTR mice at day 14 post Treg depletion. MCMV E1 was detected by quantitative PCR, and data expressed as genome copy number per 100 ng genomic DNA as described in Materials and Methods (mean+SEM); p = 0.36.
(PDF)

S8 Fig IFN-γ production upon Treg depletion in the SG. Single cell suspensions were generated from the SGs of MCMV infected mice (day7 post Treg depletion). Cells were stained for CD4, Foxp3 and IFN-γ following stimulation with or without PMA and ionomycin for 5 hours, in the presence of brefeldinA. Bar graph shows the average of frequency of IFN-γ+ in Foxp3- CD4+ (mean+SEM) in the SG. WT C57BL/6 (N = 5). Foxp3DTR (N = 5). Statistical analysis, *p ≤ 0.05 (Student’s t test).
(PDF)
