# Supplementary material for: Tissue-specific control of latent CMV reactivation by regulatory T cells
Source: PLoS Pathog. 2017 Aug 10;13(8):e1006507. doi: 10.1371/journal.ppat.1006507 (PMC5552023; doi:10.1371/journal.ppat.1006507)
Supplement: S1 Table — Table shows the number of mice with positive MCMV titers (replicating virus) in the spleen, lung, liver, pancreas and salivary gland within the three groups: Naïve, WT control and Foxp3DTR, 8 months post MCMV infection. Titers were quantified via plaque assay before Treg depletion, indicated here as Day0. 0/number of mice in each group indicates absence of actively replicating virus and confirms the establishment of latency in all tissues. (PDF) [file ppat.1006507.s001.pdf]

Day(0)	Naïve	WT MCMV	Foxp3^{-DTR} MCMV
Spleen	0/2	0/3	0/6
Lung	0/2	0/3	0/6
Liver	0/2	0/3	0/6
Pancreas	0/2	0/3	0/6
Salivary Gland	0/2	0/3	0/6

S1 Table. Establishment of latent MCMV infection. Table shows the number of mice with positive MCMV titers (replicating virus) in the spleen, lung, liver, pancreas and salivary gland within the three groups: Naïve, WT control and Foxp3^{-DTR}, 8 months post MCMV infection. Titers were quantified via plaque assay before Treg depletion, indicated here as Day0. 0/number of mice in each group indicates absence of actively replicating virus and confirms the establishment of latency in all tissues**.**
