# Supplementary material for: Tissue-specific control of latent CMV reactivation by regulatory T cells
Source: PLoS Pathog. 2017 Aug 10;13(8):e1006507. doi: 10.1371/journal.ppat.1006507 (PMC5552023; doi:10.1371/journal.ppat.1006507)
Supplement: S2 Table — Table shows the number of mice with positive MCMV titers (replicating virus) in the spleen within the two groups: WT control and Foxp3DTR, 8 months post MCMV infection. Titers were quantified by plaque assay 7 days after Treg depletion, indicated here as Day7. 0/number of mice in each group indicates absence of actively replicating virus and confirms the establishment of latency. (N = 9/group). (PDF) [file ppat.1006507.s002.pdf]

Day (7)	WT MCMV	$Foxp3^{-DTR}$ MCMV
Spleen	0/9	0/9

S2 Table. Absence of actively replicating virus in the spleen in controls and Foxp3-DTR mice 7 days post Treg depletion. Table shows the number of mice with positive MCMV titers (replicating virus) in the spleen of either C57BL/6 WT control and $Foxp3^{-DTR}$ mice 8 months post MCMV infection. Titers were quantified via plaque assay 7 days after Treg depletion, indicated here as Day7. 0/number of mice in each group indicates absence of actively replicating virus and confirms the establishment of latency. (N=9/group).
