# Supplementary material for: Tissue-specific control of latent CMV reactivation by regulatory T cells
Source: PLoS Pathog. 2017 Aug 10;13(8):e1006507. doi: 10.1371/journal.ppat.1006507 (PMC5552023; doi:10.1371/journal.ppat.1006507)
Supplement: S1 Fig — Splenocytes were isolated from naïve (9.5months old), or aged matched MCMV-latently infected mice (8months p.i.). Cells were stained for CD4 and Foxp3 and analyzed by flow cytometry. Graph shows the number of Foxp3+ cells in total CD4+ cells. Naïve (N = 4), WT MCMV infected (N = 6). (PDF) [file ppat.1006507.s003.pdf]

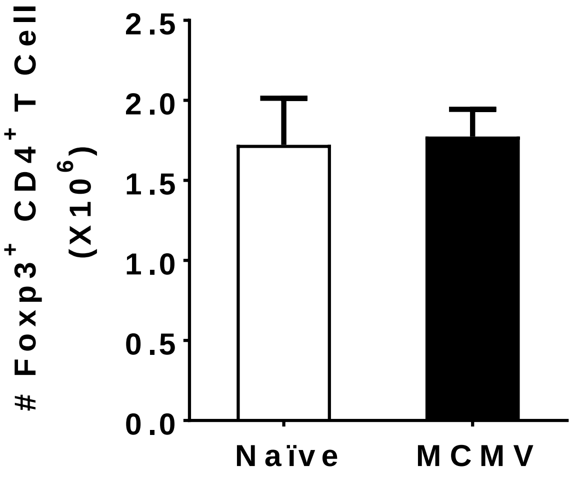

S1 Fig. Treg in the spleen during latent MCMV. Splenocytes were isolated from naïve (9.5 months), or aged matched MCMV-latently infected mice (8 months p.i.). Cells were stained for CD4 and Foxp3 and analyzed by flow cytometry. Graph shows the number of Foxp3+ cells in total CD4+ cells. Naïve (N=4), WT MCMV infected (N=6).
