# Supplementary material for: Tissue-specific control of latent CMV reactivation by regulatory T cells
Source: PLoS Pathog. 2017 Aug 10;13(8):e1006507. doi: 10.1371/journal.ppat.1006507 (PMC5552023; doi:10.1371/journal.ppat.1006507)
Supplement: S2 Fig — 5–6 week old WT C57BL/6 and Foxp3DTR mice were inoculated with 1× 10^6^ pfu of MCMV. 8 months post-MCMV infection, splenocytes were isolated from infected mice. Cells were stained for IE3, m139, M38 (CD8 T cell) tetramers day 0 (-DT) and analyzed with flow cytometry. A) Graph shows the total number of IE3-, m139- and M38-specific CD8 T from WT C57BL/6 (white bars, N = 3) and Foxp3DTR (black bars, N = 6) mice. 5–6 week old C57BL/6 and Foxp3DTRmice were inoculated with1× 106 pfu of MCMV. 8 months post-MCMV infection, both groups were injected with Diphtheria toxin (DT) on day 0, 3, 6 and sacrificed on day 7. Spleen cells were analyzed by flow cytometry. B) Bar graphs show the average frequency and absolute number of CD4+ Foxp3+Treg in the spleen (mean+SEM). WT C57BL/6 (N = 11). Foxp3DTR (N = 10). Spleen cells isolated from the two groups were stained with MCMV CD8-specific tetramers and then surface stained for expression of KLRG-1 and CD127 and intra-cellular expression of Ki67. C) Bar graph shows the total numbers of effector subpopulations within gated m139-specific CD8 T cells (mean+SEM). WT C57BL/6 (N = 6), Foxp3DTR (N = 6). D) Bar graphs show the frequency and absolute number of Ki67+ cells within M45-, IE3-, m139- and M38-specific CD8 T (mean+SEM). WT C57BL/6 (N = 6), Foxp3DTR(N = 6). Statistical analysis, *p ≤ 0.05, **p ≤ 0.01, ***p ≤ 0.001 (Student’s t-test). (PDF) [file ppat.1006507.s004.pdf]

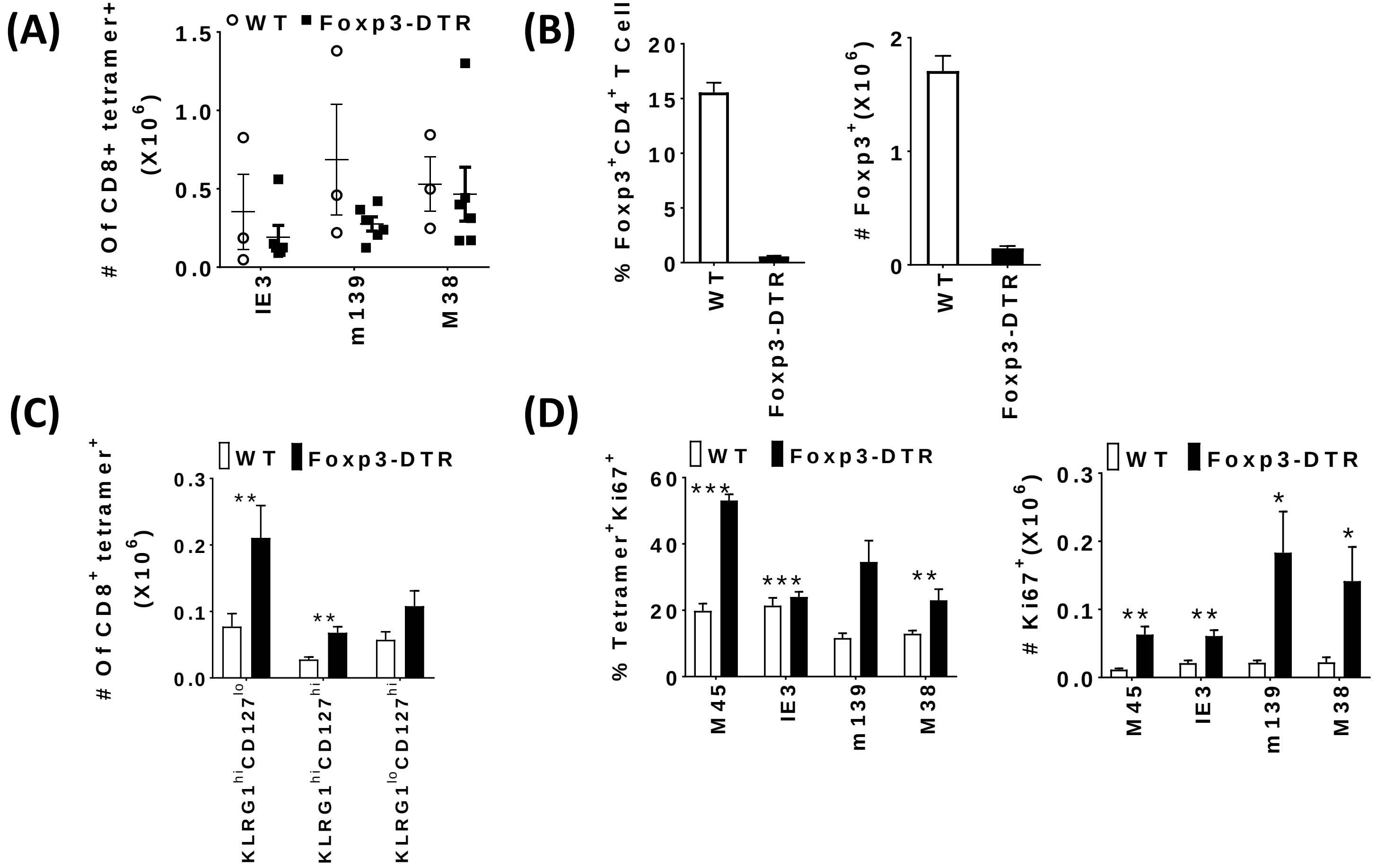

S2 Fig. Highly activated, proliferating MCMV-specific CD8+ T cells in the spleen post Treg depletion. 5-6 week old C57BL/6 and Foxp3^{DTR} mice were inoculated with1× 10⁶ pfu of MCMV. 8 months post-MCMV infection, splenocytes were isolated from infected mice. Cells were stained for IE3, m139, M38 (CD8 T cell) tetramers day 0 (-DT) and analyzed with flow cytometry. A) Graph shows the total number of IE3-, m139- and M38-specific CD8 T from WT C57BL/6 (white bars, N=3) and Foxp3^{DTR} (black bars, N=6) mice. 5-6 week old C57BL/6 and Foxp3^{DTR} mice were inoculated with1× 10⁶ pfu of MCMV. 8 months post-MCMV infection, both groups were injected with Diphtheria toxin (DT) on day 0, 3, 6 and sacrificed on day 7. Spleen cells were analyzed by flow cytometry. B) Bar graphs show the average frequency and absolute number of CD4+ Foxp3+Treg in the spleen (mean+SEM). C57BL/6 (N=11). Foxp3^{DTR} (N=10). Spleen cells isolated from the two groups were stained with MCMV CD8-specific tetramers and then surface stained for expression of KLRG-1 and CD127 and intra-cellular expression of Ki67. C) Bar graph shows the total number of effector subpopulations within gated m139-specific CD8 T cells (mean+SEM). WT C57BL/6 (N=6), Foxp3^{DTR}(N=6). D) Bar graphs show the frequency and absolute number of Ki67+ cells within M45-, IE3-, m139- and M38-specific CD8 T (mean+SEM). WT C57BL/6 (N=6), Foxp3^{DTR} (N=6).Statistical analysis, **p ≤ 0.05, **p ≤ 0.01, ***p ≤ 0.001 (*Student's*t-test).*
