# Supplementary material for: Tissue-specific control of latent CMV reactivation by regulatory T cells
Source: PLoS Pathog. 2017 Aug 10;13(8):e1006507. doi: 10.1371/journal.ppat.1006507 (PMC5552023; doi:10.1371/journal.ppat.1006507)
Supplement: S3 Fig — Genomic DNA was isolated from the spleens of WT C57BL/6 and DTR mice at day 7 post treg depletion. MCMV E1 was detected by quantitative PCR, and data expressed as genome copy number per 100 ng genomic DNA as described in Materials and Methods. Results are pooled from 4 independent experiments (WT, N = 20 and DTR, N = 22) and show the mean+SEM. The average reduction in viral load in DTR mice in 4 independent experiments was 62.7% ± 9.4 (one sample t-test p<0.007). (PDF) [file ppat.1006507.s005.pdf]

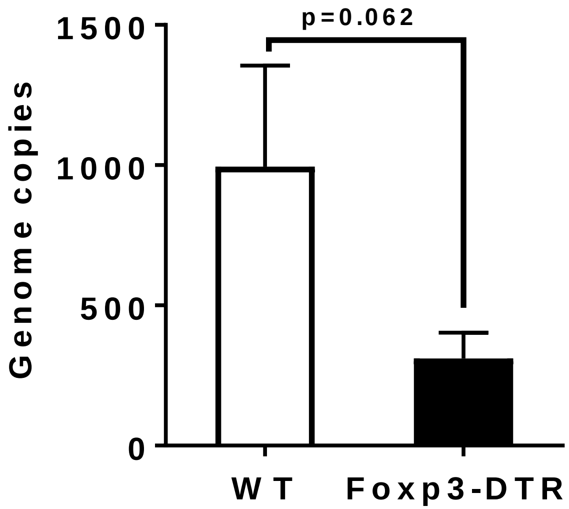

S3 Fig. MCMV viral load in the spleen. Genomic DNA was isolated from the spleens of WT C57BL/6 and DTR mice at day 7 post treg depletion. MCMV E1 was detected by quantitative PCR, and data expressed as genome copy number per 100 ng genomic DNA as described in Materials and Methods. Results are pooled from 4 independent experiments (WT, N=20 and DTR, N=22) and show the mean+SEM. The average reduction in viral load in DTR mice in 4 independent experiments was 62.7% ± 9.4 (one sample t-test p<0.007).
