# Supplementary material for: Tissue-specific control of latent CMV reactivation by regulatory T cells
Source: PLoS Pathog. 2017 Aug 10;13(8):e1006507. doi: 10.1371/journal.ppat.1006507 (PMC5552023; doi:10.1371/journal.ppat.1006507)
Supplement: S4 Fig — 5–6 week old WT C57BL/6 (white) and Foxp3DTR (black) mice were inoculated with 1× 10^6^ pfu of MCMV (N = 8/group). 9 months post-MCMV infection, both groups were injected with Diphtheria toxin (DT) on day 0, 3 and sacrificed on day4. A) Bar graph shows the percentage of mice positive for virus reactivation of naïve (UI) and MCMV infected mice in the SGs day4 post Treg depletion with the numbers of mice in each group shown above the bars. The presence of replicating virus was detected by plaque assay. B) Bar graph shows viral titers of individually homogenized SGs of naïve and MCMV infected mice Day4 post Treg depletion (mean+SEM). (PDF) [file ppat.1006507.s006.pdf]

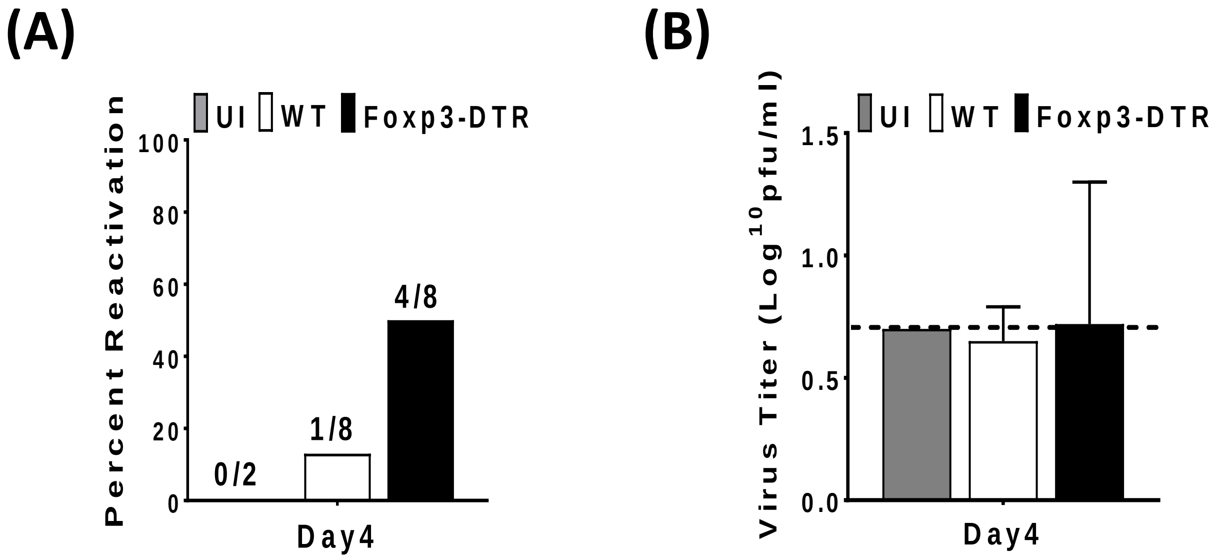

S4 Fig. Early MCMV viral reactivation post Treg depletion in the SG. 5-6 week old C57BL/6 (white) and Foxp3^{DTR} (black) mice were inoculated with 1× 10⁶ pfu of MCMV (N=8/group). 9 months post-MCMV infection, both groups were injected with Diphtheria toxin (DT) on day 0, 3 and sacrificed on day4. A) Bar graph shows the percentage of mice positive for virus reactivation in the SGs day4 post Treg depletion with the numbers of mice in each group shown above the bars. The presence of replicating virus was detected by plaque assay. B) Bar graph shows viral titers of individually homogenized SGs of naïve and MCMV infected mice Day4 post Treg depletion (mean+SEM).
