# Supplementary material for: Tissue-specific control of latent CMV reactivation by regulatory T cells
Source: PLoS Pathog. 2017 Aug 10;13(8):e1006507. doi: 10.1371/journal.ppat.1006507 (PMC5552023; doi:10.1371/journal.ppat.1006507)
Supplement: S5 Fig — 5–6 weeks old WT C57BL/6 and Foxp3DTR mice were inoculated with1× 106 pfu of MCMV. 8 months post-MCMV infection, both groups were injected with Diphtheria toxin (DT) on day 0, 3, 6 and sacrificed on day 7. A single cell suspension from the SGs of all MCMV infected mice was analyzed by flow cytometry. A) Bar graphs show the average frequency and absolute number of CD4+ Foxp3+ Treg in the SG upon DT administration (mean+SEM). WT C57BL/6 (N = 7) Foxp3DTR (N = 8). B) Bar graph shows the average frequency of tissue resident memory (CD103+CD69+) cells in the SG within the total M38-specific CD8 T cells (mean+SEM). C57BL/6 (N = 4). Foxp3DTR (N = 5). Statistical analysis, *p ≤ 0.05, **p ≤ 0.01, ***p ≤ 0.001 (Student’s t-test). (PDF) [file ppat.1006507.s007.pdf]

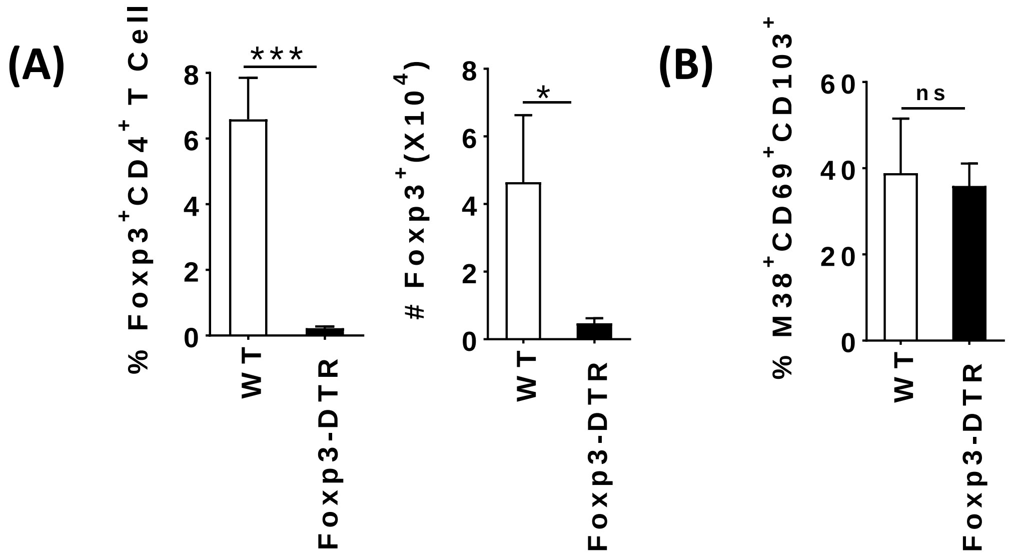

S5 Fig. Efficiency of Treg depletion in the SG. 5-6 weeks old C57BL/6 and Foxp3^{DTR} mice were inoculated with1× 10^6 pfu of MCMV. 8 months post-MCMV infection, both groups were injected with Diphtheria toxin (DT) on day 0, 3, 6 and sacrificed on day 7. A single cell suspension from the SGs of naïve and MCMV infected mice was analyzed by flow cytometry. A) Bar graphs show the average frequency and absolute number of CD4+ Foxp3+ Treg in the SG upon DT administration (mean+SEM). C57BL/6 (N=7) Foxp3^{DTR} (N=8). B) Bar graph shows the average frequency of tissue resident memory (CD103+CD69+) cells in the SG within the total M38-specific CD8 T cells (mean+SEM). C57BL/6 (N=4). Foxp3^{DTR} (N=5). Statistical analysis, *p ≤ 0.05, **p ≤ 0.01, ***p ≤ 0.001 (Student's t-test).
