# Supplementary material for: Tissue-specific control of latent CMV reactivation by regulatory T cells
Source: PLoS Pathog. 2017 Aug 10;13(8):e1006507. doi: 10.1371/journal.ppat.1006507 (PMC5552023; doi:10.1371/journal.ppat.1006507)
Supplement: S6 Fig — 5–6 weeks old WT C57BL/6 (white bars) and Foxp3DTR (black bars) mice were inoculated with 1× 10^6^ pfu of MCMV. 8 months post-MCMV infection, both groups were injected with Diphtheria toxin (DT) on day 0, 3, 6 and sacrificed on day 7. A) Single cell suspensions were generated from the spleen of MCMV infected WT C57BL/6 (N = 11) and Foxp3DTR (N = 10) mice (day7) post Treg depletion. Cells were stained for TCRß, CD4, CD8, Foxp3 and IL-10 following stimulation with or without PMA and ionomycin for 5 hours, in the presence of brefeldinA in the final 4hrs. Bar graph shows the average of frequency of IL-10+ in Foxp3- CD4+ TCRβ+ and CD8+ TCRβ+ cells and TCR-β− cells (mean+SEM). B) Graph shows the average normalized IL-10 mRNA level in spleen of MCMV-infected WT C57BL/6 (N = 10) and Foxp3DTR (N = 9) mice (day7) post Treg depletion (mean+SEM). Statistical analysis, *p ≤ 0.05, **p ≤ 0.01 (Student’s t test). (PDF) [file ppat.1006507.s008.pdf]

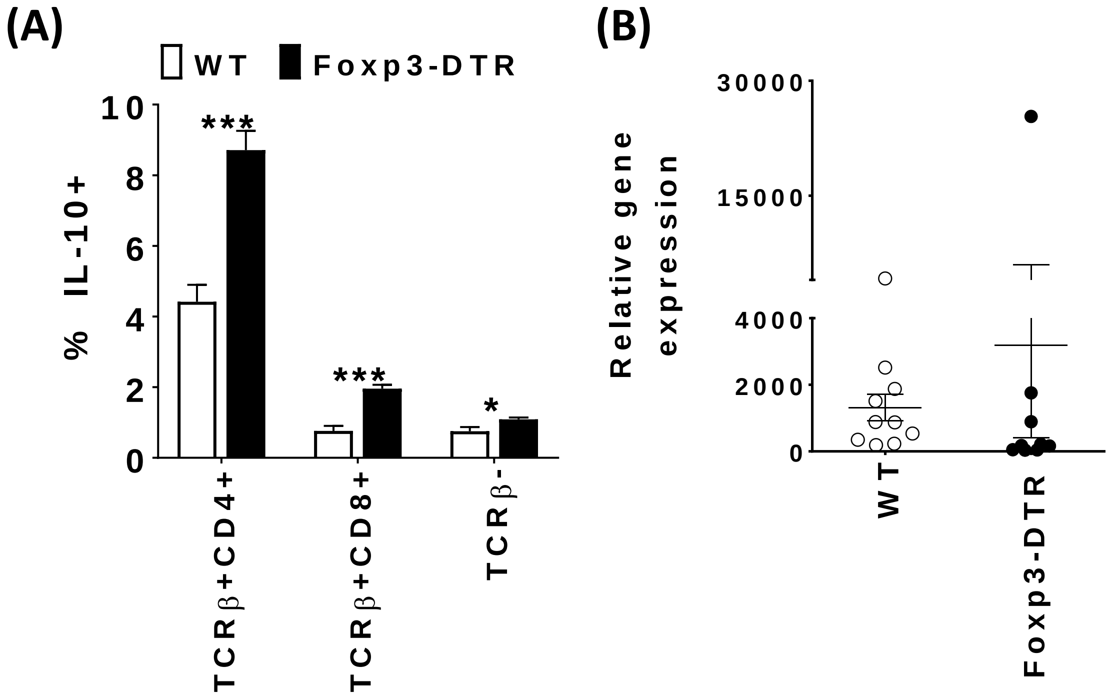

S6 Fig. Treg suppress CD4+ Foxp3- IL-10+ cells in the spleen. 5-6 weeks old WT C57BL/6 (white bars) and Foxp3^{DTR} (black bars) mice were inoculated with 1× 10^6 pfu of MCMV. 8 months post-MCMV infection, both groups were injected with Diphtheria toxin (DT) on day 0, 3, 6 and sacrificed on day 7. A) Single cell suspensions were generated from the spleen of MCMV infected C57BL/6 (N=11) and Foxp3^{DTR} (N=10) mice (day7) post Treg depletion. Cells were stained for TCRß, CD4, CD8, Foxp3 and IL-10 following stimulation with or without PMA and ionomycin for 5 hours, in the presence of brefeldinA in the final 4hrs. Bar graph shows the average of frequency of IL-10+ in Foxp3- CD4+TCRβ+ and CD8+TCRβ+ cells and TCR-β− cells (mean+SEM). B) Graph shows the average normalizedIL-10 mRNA level in spleen of MCMV-infected C57BL/6 (N=10) and Foxp3^{DTR} (N=9) mice (day7) post Treg depletion (mean+SEM). Statistical analysis, *$p \le 0.05$, **$p \le 0.01$, ***$p \le 0.001$ (Student's *t* test).
