# Supplementary material for: Tissue-specific control of latent CMV reactivation by regulatory T cells
Source: PLoS Pathog. 2017 Aug 10;13(8):e1006507. doi: 10.1371/journal.ppat.1006507 (PMC5552023; doi:10.1371/journal.ppat.1006507)
Supplement: S7 Fig — 5–6 week old WT C57BL/6 (white) and Foxp3DTR (black) mice were inoculated with 1× 10^6^ pfu of MCMV (N = 8/group). 5 months post-MCMV infection, both groups were injected with Diphtheria toxin (DT) on day 0, 3, 6, 9,12 and sacrificed on day 14. A) Bar graph shows the percentage of mice positive for virus replication in the spleen day14 quantified by plaque assay post Treg depletion with the numbers of mice in each group shown above the bars. Viral titers were 18.6 pfu/ml +/- 15.5 in WT C57BL/6 mice and 2.4 pfu/ml +/- 2.24 in FoxP3-DTR mice; p = 0.31. B) Genomic DNA was isolated from the spleens of WT C57BL/6 and DTR mice at day 14 post Treg depletion. MCMV E1 was detected by quantitative PCR, and data expressed as genome copy number per 100 ng genomic DNA as described in Materials and Methods (mean+SEM); p = 0.36. (PDF) [file ppat.1006507.s009.pdf]

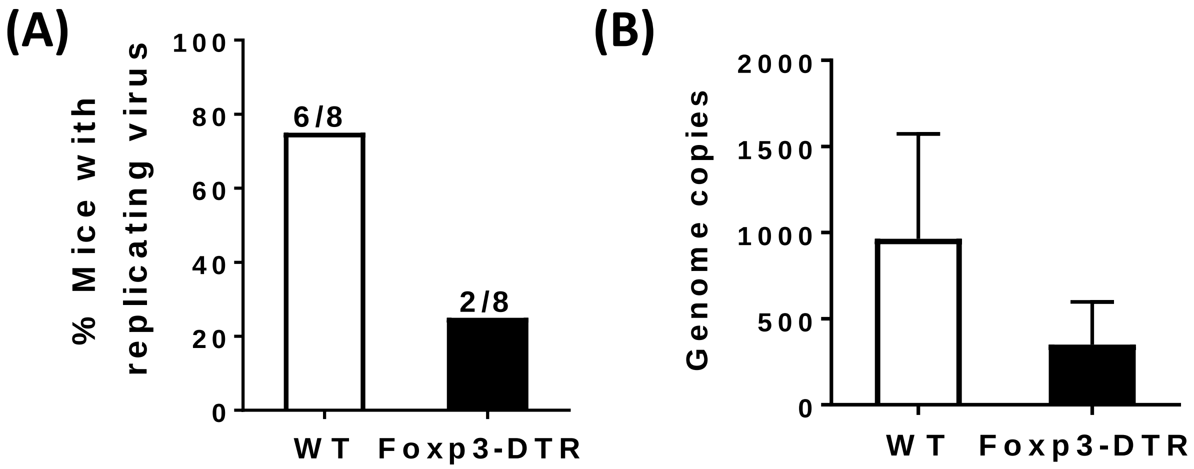

S7 Fig. Treg promote MCMV replication in the spleen. 5-6 week old C57BL/6 (white) and Foxp3^{DTR} (black) mice were inoculated with 1× 10^6 pfu of MCMV (N=8/group). 5 months post-MCMV infection, both groups were injected with Diphtheria toxin (DT) on day 0, 3, 6, 9,12 and sacrificed on day 14. A) Bar graph shows the percentage of mice positive for virus replication in the spleen day14 quantified via plaque assay post Treg depletion with the numbers of mice in each group shown above the bars. Viral titers were 18.6 pfu/ml +/-15.5 in C57BL/6 mice and 2.4 pfu/ml +/- 2.24 in FoxP3-DTR mice; p=0.31. B) Genomic DNA was isolated from the spleens of WT C57BL/6 and DTR mice at day 14 post treg depletion. MCMV E1 was detected by quantitative PCR, and data expressed as genome copy number per 100 ng genomic DNA as described in Materials and Methods (mean+SEM); p=0.36.

.
