# Supplementary material for: Tissue-specific control of latent CMV reactivation by regulatory T cells
Source: PLoS Pathog. 2017 Aug 10;13(8):e1006507. doi: 10.1371/journal.ppat.1006507 (PMC5552023; doi:10.1371/journal.ppat.1006507)
Supplement: S8 Fig — Single cell suspensions were generated from the SGs of MCMV infected mice (day7 post Treg depletion). Cells were stained for CD4, Foxp3 and IFN-γ following stimulation with or without PMA and ionomycin for 5 hours, in the presence of brefeldinA. Bar graph shows the average of frequency of IFN-γ+ in Foxp3- CD4+ (mean+SEM) in the SG. WT C57BL/6 (N = 5). Foxp3DTR (N = 5). Statistical analysis, *p ≤ 0.05 (Student’s t test). (PDF) [file ppat.1006507.s010.pdf]

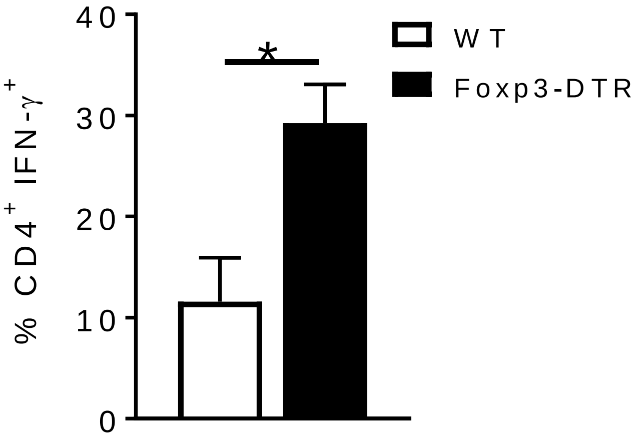

S8 Fig. IFN-γ production upon Treg depletion in the SG. Single cell suspensions were generated from the SGs of MCMV infected mice (day7 post Treg depletion). Cells were stained for CD4, Foxp3 and IFN-γ following stimulation with or without PMA and ionomycin for 5 hours, in the presence of brefeldinA. Bar graph shows the average of frequency of IFN-γ+ in Foxp3- CD4+ in the SG (mean+SEM). C57BL/6 (N=5). Foxp3^{DTR} (N=5). Statistical analysis, *$p \leq 0.05$ (Student's *t* test).
